# Quantitative Estimation of Organic Pollution in Inland Water Using Sentinel-2 Multispectral Imager

**DOI:** 10.3390/s25092737

**Published:** 2025-04-26

**Authors:** Jiayi Li, Ruru Deng, Yu Guo, Cong Lei, Zhenqun Hua, Junying Yang

**Affiliations:** 1 School of Geography and Planning, Sun Yat-sen University, Guangzhou 510006, China; lijy286@mail2.sysu.edu.cn (J.L.); guoy87@mail2.sysu.edu.cn (Y.G.); leic@mail2.sysu.edu.cn (C.L.); huazhq@mail2.sysu.edu.cn (Z.H.); yangjy257@mail2.sysu.edu.cn (J.Y.); 2 Guangdong Engineering Research Center of Water Environment Remote Sensing Monitoring, Guangzhou 510275, China; 3 Southern Marine Science and Engineering Guangdong Laboratory (Zhuhai), Zhuhai 519082, China; 4 Guangdong Provincial Key Laboratory of Urbanization and Geo-Simulation, Guangzhou 510275, China

**Keywords:** water quality remote sensing, organic pollution, radiative transfer model, Sentinel-2, Feilaixia basin

## Abstract

Organic pollution poses a significant threat to water security, making the monitoring of organic pollutants in water environments essential for the protection of water resources. Remote sensing technology, with its wide coverage, continuous monitoring capability, and cost-efficiency, overcomes the limitations of traditional methods, which are often time-consuming, labor-intensive, and spatially restricted. As a result, it has become an effective tool for monitoring organic pollution in water environments. In this study, we propose a physically constrained remote sensing algorithm for the quantitative estimation of organic pollution in inland waters based on radiative transfer theory. The algorithm was applied to the Feilaixia Basin using Sentinel-2 data. Accuracy assessment results demonstrate good performance in the quantitative assessment of organic pollution, with a coefficient of determination (R^2^) of 0.79, a mean absolute percentage error (MAPE) of 13.03%, and a root mean square error (RMSE) of 0.39 mg/L. Additionally, a seasonal variation map of organic pollutant concentrations in the Feilaixia Basin was generated, providing valuable scientific support for regional water quality monitoring and management.

## 1. Introduction

With the rapid development of the economy and society, water pollution has become a significant challenge both in China and globally. The proposal of sustainable development strategies has made water environment protection an important task for governments. However, water security continues to be threatened by the deterioration of water quality [1,2,3,4]. The prerequisite for protecting the water environment is effective monitoring. Traditional monitoring methods, such as the determination of chemical oxygen demand (COD), total organic carbon (TOC), and five-day biochemical oxygen demand (BOD_5_), typically involve in situ sampling, sample preservation, and laboratory-based analysis. For instance, COD is commonly measured through chemical titration or spectrophotometry after oxidation with potassium dichromate or permanganate; TOC is determined using high-temperature catalytic combustion; and BOD_5_ requires a five-day incubation period to evaluate microbial oxygen consumption. While these methods are well-established and provide accurate point-based results, they are time-consuming and labor-intensive, and their application is limited in terms of spatial coverage and frequency, making them inadequate for large-scale or continuous monitoring [5,6,7,8]. In contrast, remote sensing monitoring offers advantages such as a broad detection range, continuous monitoring capabilities, and cost-effectiveness [9,10,11], overcoming the limitations of traditional techniques and being widely applied in water environment monitoring [12,13,14,15].

Organic pollution, a key indicator in water quality evaluation, is closely tied to water security. Severe organic pollution can lead to the overgrowth of heterotrophic microorganisms, causing water discoloration and unpleasant odors—commonly referred to as “black-odorous water” [16]—which ultimately results in a loss of water resources. In addition, inorganic nutrients such as phosphates can contribute to unpleasant odors by promoting eutrophication and excessive algal growth, with odors released during algal decay. It has been confirmed that the molecular weight of organic matter in surface water affects the binding of Cu^2+^, consequently influencing the distribution, migration, and transformation of heavy metal pollutants [17]. This poses a risk to human health through transmission and accumulation in the food chain. Moreover, due to the diverse composition of organic micropollutants in water, even advanced drinking water treatment technologies cannot fully eliminate the risk of pollutant accumulation [18], thus posing a potential threat to drinking water safety. Organic pollution in water environments not only detracts from urban aesthetics but also presents both direct and indirect risks to human health. Therefore, close attention to organic pollution is critical for water environment protection.

In remote sensing monitoring of organic pollution in water environments, commonly used methods can be categorized into empirical, analytical, and semi-empirical/semi-analytical methods [9,11,19]. Empirical methods aim to establish statistical relationships between measured water quality parameters and the reflectance of remotely sensed images, including techniques such as linear and multiple linear regression, curve fitting, and nonlinear regression [20,21,22,23]. In recent years, with the development of artificial intelligence (AI), intelligent algorithms such as Artificial Neural Network (ANN), Support Vector Machine (SVM), and Random Forest (RF) have also been successfully applied to the remote sensing monitoring of organic pollution in water environments [24,25,26,27]. For example, EI Din et al. developed a back-propagation neural network (BPNM) inversion algorithm for high-precision optical and non-optical surface water quality parameters, including turbidity, Total Suspended Solids (TSS), chemical oxygen demand (COD), Biological Oxygen Demand (BOD), and Dissolved Oxygen (DO), based on Landsat 8 satellite data [28]. Although these methods generally offer high accuracy, they require large datasets and are difficult to generalize to different environments.

Analytical methods, on the other hand, are based on bio-optical and radiative transfer models that simulate the transmission of light through the atmosphere and water to determine the relationship between the optical properties of water quality parameters and the reflectance of remotely sensed images [29,30,31]. Arabi et al. used a radiative transfer model to obtain a 15-year diurnal variation of water constituent concentrations in the Dutch Wadden Sea from multi-sensor satellite images and in situ hyperspectral measurements [32]. These methods, rooted in physical mechanisms, have clear physical meanings, do not rely on measured data, and are not restricted to specific regions, making them applicable to various environments. However, they are more complex and computationally intensive due to their reliance on physical drivers.

Semi-empirical/semi-analytical methods, also known as hybrid methods, combine empirical and analytical methods. They derive physical mechanisms and use a small amount of measured data to select the optimal band combination for estimating water quality parameters [33,34,35,36]. For instance, Cao et al. used a QAA-CDOM semi-analytical model to invert the concentration of colored organic dissolved matter (CDOM) in Lake Ebinur from May to October during 2018 to 2022, achieving good accuracy [37]. These methods have some physical significance and require less measurement data, enhancing their generalizability. However, they still face certain spatial and temporal limitations in practical applications.

Despite the growing application of remote sensing in water quality monitoring, most existing studies on organic pollution rely heavily on empirical or semi-empirical models, which are often site-specific and lack physical interpretability. In this context, this study aims to develop a remote sensing method for the quantitative estimation of organic pollution in inland waters and to obtain key optical parameters of organic pollutants. The main objectives of the research are (1) to investigate the spectral characteristics of organic pollutants in water bodies and determine its inherent optical properties; (2) to develop and validate a remote sensing inversion algorithm for organic pollutant concentrations that effectively separates other water quality components; and (3) to map the distribution of organic pollutant concentrations in typical basins and analyze their spatial distribution patterns and trends. Using Sentinel-2 data, the proposed algorithm was applied to the Feilaixia Basin, achieving accurate estimation of organic pollutant levels. This research provides valuable scientific support for inland water environment monitoring and water quality management.

## 2. Materials and Methods

### 2.1. Study Area

The Beijiang River is a major tributary of the Pearl River system, one of the three largest water systems in China. It extends for 582 km and covers a watershed area of 43,000 square kilometers, with 92% of its length flowing through Guangdong Province [38]. The Feilaixia Basin, located in the middle reaches of the Beijiang River, accounts for 73% of the Beijiang River basin [39], making it a significant part of the system. The basin is characterized by predominantly mountainous and hilly terrain, with higher elevations in the north and lower elevations in the south. It lies within the subtropical monsoon climate zone [40], experiencing cloudy and rainy springs; hot and humid summers; frequent hot thunderstorms and typhoon rains in autumn; and cold, dry winters. These climatic characteristics result in strong seasonal variations in precipitation and runoff, which significantly affect hydrological processes and pollutant transport within the basin.

**Figure 1:**
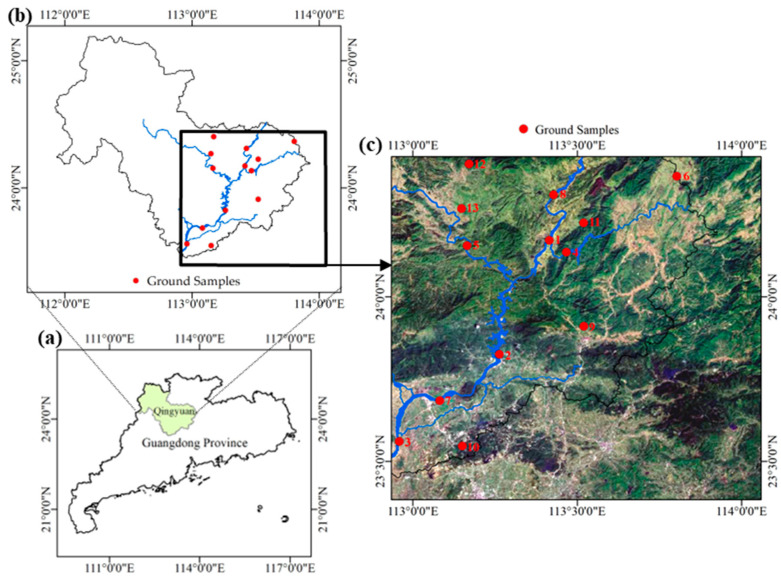
Study area: (**a**) Qingyuan City, Guangdong Province, China, (**b**) the location of study area, (**c**) the location of ground samples.

The core mainstream of the Feilaixia Basin is the middle section of the Beijiang River, with major tributaries including the Wengjiang, Lianjiang, Pajiang, and Binjiang Rivers, running from upstream to downstream, as shown in Figure 1b. Additionally, the midstream of the basin features the Feilaixia Water Conservancy Hub, a key hydraulic project in Guangdong Province that serves functions such as flood control, navigation, and power generation [41]. The Feilaixia Basin traverses the core economic zone of Qingyuan City, where urban development, industrial production, and agricultural activities are highly concentrated. These sectors make important contributions to the local economy and regional GDP. However, they also exert increasing pressure on the water environment through pollutant discharge and intensive land use. As the basin merges into the Pearl River Delta, its water quality directly affects the water security of downstream cities such as Guangzhou and Foshan [42], making it a vital component of the comprehensive water quality management efforts in the Beijiang River basin.

Overall, the main stream of the study area maintains good water quality, with wide and deep river sections. However, water quality in various tributaries differs significantly due to urban clusters and industrial and agricultural activities along their courses. Therefore, monitoring organic pollution in the Feilaixia Basin provides scientific data for protecting the water security of downstream cities and preserving the ecological environment within the basin.

### 2.2. In Situ Measurement Data

The in situ measurement data used in this study were provided by the Guangdong Hydrological Bureau, covering a total of 13 monitoring stations, as shown in Figure 1c. These stations are primarily located in ecologically significant rivers or reservoirs, including major rivers, key tributaries, and important drinking water sources. Samples were collected, and measurements were taken directly from the target water bodies. The monitored water quality parameters include the permanganate index (COD_Mn_), ammonia nitrogen (NH_3_-N), total phosphorus (TP), total nitrogen (TN), volatile phenol (ArOH), and chlorophyll a (Chl-a).

All water quality measurements were conducted in accordance with the Chinese National Environmental Protection Standard HJ 915-2017 [43], Technical Specification for Automatic Monitoring of Surface Water. Among the monitored parameters, COD_Mn_ is a mandatory indicator for surface water quality monitoring stations. It was measured using either the electrode method or spectrophotometry, with a detection limit of 1 mg/L. For this study, the synchronized daily average values of COD_Mn_ were selected as the in situ measurement data.

### 2.3. Satellite Data Acquisition and Processing

#### 2.3.1. Satellite Data

Sentinel-2 data are widely used in the remote sensing monitoring of water environments due to its high resolution, multispectral capabilities, and short revisit period [44,45,46]. Sentinel-2 is a high-resolution multispectral imaging satellite launched by the European Space Agency (ESA) in 2015, consisting of two satellites: Sentinel-2A and Sentinel-2B. The Sentinel-2 satellites carry a multispectral imager (MSI) that covers 13 spectral bands: 4 bands with a spatial resolution of 10 m, 6 bands with a resolution of 20 m, and 3 bands with a resolution of 60 m, as shown in Table 1. The satellites have a 5-day revisit period and an image swath width of 290 km.

In this study, Sentinel-2 MSI Level-1C image were used, acquired on the following dates: 5 March 2023; 29 May 2023; 21 September 2023; and 25 December 2023. These four images were selected to represent typical seasonal conditions—spring, summer, autumn, and winter—and to capture the corresponding variations in water quality. To ensure data quality, the selected images were required to have cloud cover of less than 10% over the study area and minimal atmospheric interference. Additionally, water level and meteorological conditions were considered to avoid anomalous events such as floods or extreme droughts that might bias the reflectance signals. The data were downloaded from Copernicus Open Access Hub [47].

#### 2.3.2. Radiometric Calibration

The DN value (*L1C_DN*) of the Sentinel-2 Level-1C image is converted to top-of-atmosphere reflectance (*ρ_TOA_*) according to Sentinel-2 MSI Level-1C Algorithm Overview [48] provided by European Space Agency:

(1) $$\rho_{\mathrm{TOA}}=\left(L1C\_\mathrm{DN}+\mathrm{RADIO}\_\mathrm{ADD}\_\mathrm{OFFSET}\right)\;/\;\mathrm{QUANTIFICATION}\_\mathrm{VALUE}$$

where *RADIO_ADD_OFFSET* is a band-dependent constant radiometric offset used to avoid the truncation of negative values. *QUANTIFICATION_VALUE* is a fixed coefficient that preserves the dynamic range of the data. These parameters can be obtained from the image’s metadata.

#### 2.3.3. Atmospheric Correction

Water bodies, with their low reflectance, are particularly sensitive to the absorption and scattering of atmospheric radiation transmission, making atmospheric correction a key part of water quality remote sensing.

The reflection signal received by the satellite sensor consists of both the ground reflectance after atmospheric attenuation and atmospheric path radiation. It is assumed that the ground behaves as a Lambertian surface and that solar radiation is isotropic.

The relationship between top-of-atmosphere reflectance and ground object reflectance (*ρ_g_*) can be expressed as follows:

(2) $$\rho_{\mathrm{TOA}}=\rho_{g}\cdot T_{u}\left(T_{d}+\frac{\tau }{2}\sec\theta \right)+\frac{\tau }{4}\sec\theta$$

where *T_u_* is the ascending atmospheric transmittance, given by $T_{u}=e^{-\;\tau \cdot \sec\varphi }$, and *T_d_* is the descending atmospheric transmittance, expressed as $T_{d}=e^{-\;\tau \cdot \sec\theta }$. Here, *φ* is the zenith angle observed by the satellite sensor, *θ* is the solar zenith angle, and *τ* represents the optical thickness of the atmosphere.

For remote sensing monitoring of water environments, atmospheric correction must fully account for the absorption and scattering effects of water vapor to restore the reflectance of water body pixels as accurately as possible. Therefore, in this study, several clear and deep water pixels were selected as “dark pixels”, and the atmospheric optical thickness of image pixels was calculated based on their relative weights compared to each dark pixel [49]. Using Equation (2), the reflectance of the planetary surface was converted to the ground object reflectance, thereby achieving atmospheric correction.

#### 2.3.4. Water Extraction

In this study, the widely used Normalized Differential Water Index (*NDWI*) [50] was employed to extract water bodies in the study area. The calculation formula is as follows:

(3) $$\mathrm{NDWI}\;=\frac{\mathrm{Green}-\mathrm{NIR}}{\mathrm{Green}+\mathrm{NIR}}$$

where *Green* is the reflectance of the green band, and *NIR* indicates the reflectance of near-infrared band.

Additionally, a near-infrared band threshold is introduced to separate water from land. The criterion for water extraction is as follows:

(4) NDWI > 0.03 ∩ NIR < 0.08

### 2.4. Organic Pollution Inversion Model

Water bodies exhibit a degree of transparency, allowing incident light to penetrate the water surface, particularly within the visible wavelength range. After being radiatively transmitted through the water, the emergent light contains information about the water body, which can be detected using remote sensing technology. This makes it possible to retrieve data on water bodies.

When electromagnetic waves penetrate the atmosphere and reach the water surface, part of the wave is directly reflected, while another portion penetrates the water. Within the water, these waves are absorbed and scattered by water components, and part of these waves are reflected from the water bottom. The scattered light from the water column and the bottom-reflected light combine to form the emergent light, referred to as off-water radiance. This radiance contains critical information about water column, including characteristics such as component types, concentrations, and water depth. The emergent light is essential for remote sensing inversion models.

Assuming uniform scattering across hemispheres, the total upward scattered radiance (*L_s_^+^*) can be expressed as

(5) $${L_{s}}^{+}=\frac{1}{2\pi \cdot \mathrm{uk}}E_{0}\cdot \beta \cdot \left[1-e^{-\left(1+\sec\theta^{\prime }\right)kH}\right]$$

where *θ′* is the angle of refraction of incident light in water; *E*_0_ is the incident light irradiance, *H* is water depth; and *k* is the water’s extinction coefficient, which is expressed as k=α+β, where *α* is the absorption coefficient and *β* is the scattering coefficient of the water.

In most cases, water bodies such as rivers and lakes are sufficiently deep such that the bottom reflection can be considered negligible. Assuming the water depth *H* approaches infinity, the off-water radiance (*L_W_*) can be expressed as:

(6) $$L_{w}=\frac{1}{2\pi \cdot \left(1+\sec\theta^{\prime }\right)k}E_{0}\cdot \beta$$

The off-water reflectance (*ρ_w_*) is then expressed as

(7) $$\rho_{w}=\frac{\beta }{2\cos\varphi^{\prime }\left(1+\sec\theta^{\prime }\right)\left(\alpha +\beta \right)}$$

where *φ*′ is the refracted observation angle in water.

The inherent optical characteristics of water bodies are primarily influenced by water molecules and various water quality components, rather than the distribution and intensity of incident light [51]. As a result, the absorption and scattering coefficients of the water body are the sums of the absorption and scattering coefficients of both water molecules and other water quality components.

(8) $$\alpha \;=\alpha_{w}+\sum C_{i}\cdot \alpha_{i}$$

(9) $$\beta =\beta_{w}+\sum C_{i}\cdot \beta_{i}$$

Thus, the off-water reflectance becomes a function of the organic pollutant concentration (*C_op_*):

(10) $$\rho_{w}=f(C_{op})$$

**Figure 2:**
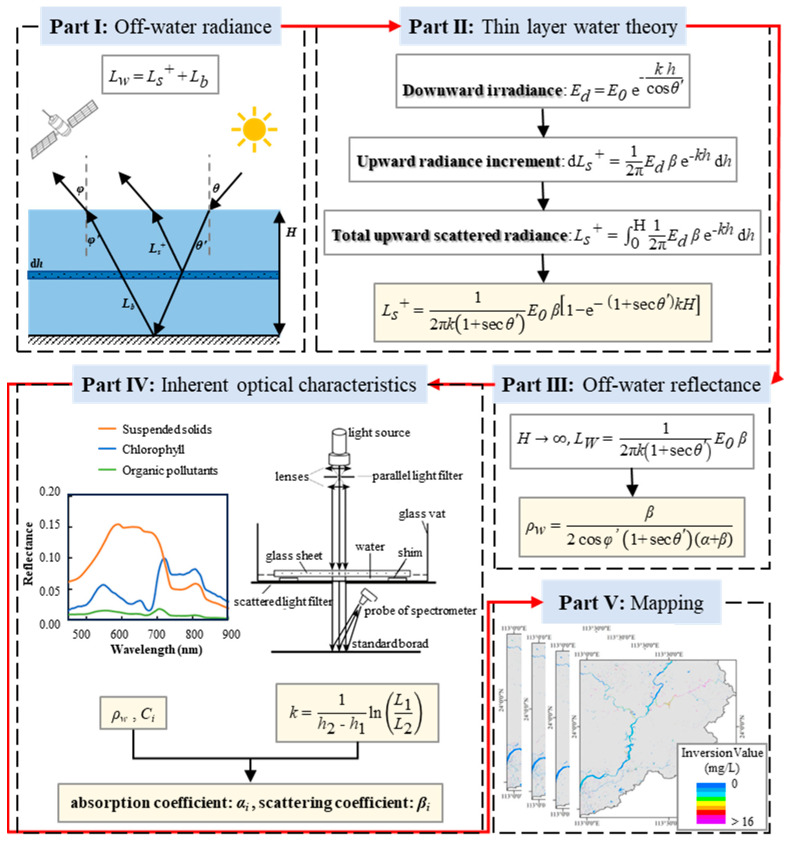
Flowchart used for quantitative estimation of organic pollutant concentration.

Figure 2 illustrates the main process used for the quantitative estimation of the organic pollutant concentration in this study.

### 2.5. Acquisition of Optical Parameters

**Figure 3:**
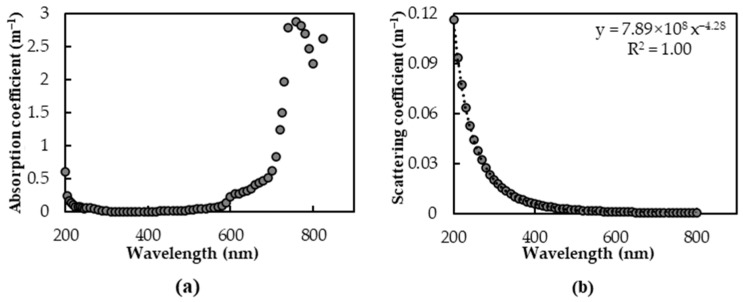
The inherent optical parameters of pure water: (**a**) absorption coefficient, (**b**) scattering coefficient.

The inherent optical parameters of the water molecule are stable. In this study, the absorption coefficient of pure water, as provided by Fewell and Trojan [52], and the scattering coefficient of pure water, as provided by Smith and Baker [53], were used as a basis, as shown in Figure 3.

According to the measurement data uncovered by Smith and Baker [53], the scattering coefficient of pure water (*β_w_*) can be expressed as

(11) $$\beta_{w}=\left(7.89\;E8\right)\lambda^{-4.28}$$

where *λ* is wavelength.

In this study, a combination of outdoor natural water spectral measurements and indoor water extinction coefficient measurements were used to obtain the absorption and scattering coefficients of each water quality component. It was assumed that one water quality component in the water is dominant while other components can be neglected. Organic pollutants were taken as an example. The scattering coefficient (*β_op_*) of organic pollutants was calculated as follows:

(12) $$\beta_{op}=\frac{2\cos\varphi^{\prime }\left(1+\frac{1}{\cos\theta^{\prime }}\right)k\cdot \rho_{w}-\beta_{w}}{C_{op}}$$

where *φ*′ and *θ*′ are calculated from the angles measured in the field. *k* is obtained from the indoor water extinction coefficient measurement experiments, and *C_op_* is the concentration of organic pollutants measured by the indoor analysis experiments.

The resulting absorption coefficient (*α_op_*) for organic pollutants is

(13) $$\alpha_{op}=\frac{k-\alpha_{w}-\beta_{w}-C_{op}\cdot \beta_{op}}{C_{op}}$$

where *α_w_* is the absorption coefficients of pure water.

### 2.6. Evaluation Index

In this study, four metrics were used to assess the accuracy of the remote sensing inversion model: relative error (*RE*), coefficient of determination (*R*^2^), mean absolute percentage error (*MAPE*), and root mean square error (*RMSE*). *R*^2^ indicates how well the regression equation fits the measured data, while *MAPE* and *RMSE* quantify the discrepancy between the remote sensing inversion data and the measured data [54]. The formulas for calculating each indicator are presented below:

(14) $$\mathrm{RE}=\frac{\left|M_{i}-I_{i}\right|}{M_{i}}$$

(15) $$R^{2}=1\;-\;\frac{\sum_{i=1}^{n}{\left(M_{i}-I_{i}\right)}^{2}}{\sum_{i=1}^{n}{\left(M_{i}-\overset{-}{M}\right)}^{2}}$$

(16) $$\mathrm{MAPE}=\frac{1}{n}\sum_{i=1}^{n}\frac{\left|M_{i}-I_{i}\right|}{M_{i}}\;\times \;100\%$$

(17) $$\mathrm{RMSE}=\sqrt{\frac{1}{n}\sum_{i=1}^{n}{\left(M_{i}-I_{i}\right)}^{2}}$$

where *n* is the number of measured samples, *M_i_* is the measured value of sample *i*, and *I_i_* is the remote sensing inversion value of sample *i*. Additionally, $\overset{-}{M}$ indicates the average of the measured values of all samples.

## 3. Results

### 3.1. Spectral Characteristics

**Figure 4:**
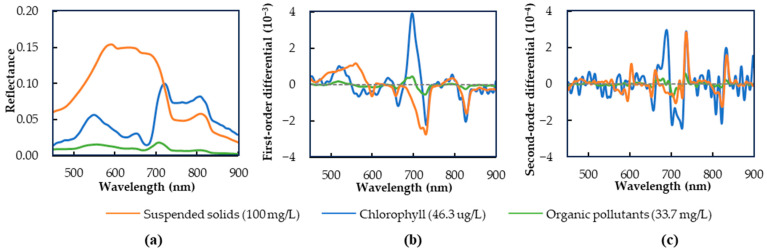
The spectrum of typical water samples with a single component: (**a**) raw spectra, (**b**) first-order differential spectra, (**c**) second-order differential spectra.

Based on the environmental characteristics of the study area, this study considers three typical water quality components in Southern China, including organic pollutants, suspended solids, and chlorophyll. To minimize interference between these components, typical water samples containing a single component were selected for spectral measurements, with their spectral curves shown in Figure 4a.

Organic pollutants, unlike the high reflectance features of suspended solids and chlorophyll, primarily affect the water’s spectrum through absorption, particularly in the visible light range. The spectra of organic pollutants are characterized by peaks and troughs in the 500–750 nm range, where reflectance is negatively correlated with the concentration of organic pollutants [55]. Specifically, spectral reflectance peaks occur near 555 nm and 710 nm, while absorption troughs are observed around 670 nm [56].

Raw spectra display the magnitude of reflectance at specific wavelengths, while differential spectra help eliminate background noise, facilitating the distinction of overlapping spectral features. First-order differential spectra highlight the rate of change in reflectance at specific wavelengths, whereas second-order differential spectra reveal the concave and convex characteristics of the original spectral curve. In this study, both first- and second-order differential spectral analyses were performed on typical single-component water samples, with the results presented in Figure 4b,c.

The first-order differential spectrum of organic pollutants transitions from positive to negative in the 545–565 nm, 700–720 nm, and 795–815 nm wavelength ranges, while the second-order differential spectrum remains negative. The original spectrum exhibits a pattern of increasing and then decreasing reflectance, forming convex-shaped peaks near 555 nm, 710 nm, and 805 nm. In contrast, within the 660–680 nm range, the spectrum reveals a trough near 670 nm. Similarly, the chlorophyll spectrum displays peaks near 550 nm, 650 nm, 720 nm, and 805 nm, with corresponding troughs near 630 nm and 675 nm. For suspended solids, spectral peaks occur near 590 nm and 805 nm.

Within the visible light range, the spectrum of suspended solids displays a significant reflectance peak near 590 nm, with the highest overall reflectance among the three components, clearly distinguishing it from the other two. In comparison, both organic pollutants and chlorophyll show similar spectral trends, but chlorophyll has a distinct peak near 650 nm with higher reflectance, differentiating it from organic pollutants. In the near-infrared range, all three components exhibit similar spectral trends, with a sharp decline in reflectance and peaks near 805 nm. However, suspended solids and chlorophyll reflect much more strongly than organic pollutants. These spectral characteristics enable the effective differentiation of organic pollutants from other water quality components.

### 3.2. Accuracy Assessment

A statistical comparison between the observed values and the remote sensing inversion values is presented in Table 2. The observed values range from 0.9 to 3.7 mg/L, with an average of 2.0 mg/L. In contrast, the remote sensing inversion values range from 0.801 to 4.020 mg/L, with an average of 2.071 mg/L. The concentration ranges and average values of the two datasets are similar, indicating minimal overall differences.

The results of the relative error (RE) calculations reveal a small overall deviation, with an average RE value of 0.13. However, sample NO.9 has a larger RE value of 1.02, making it the sample with the largest deviation in the dataset.

**Figure 5:**
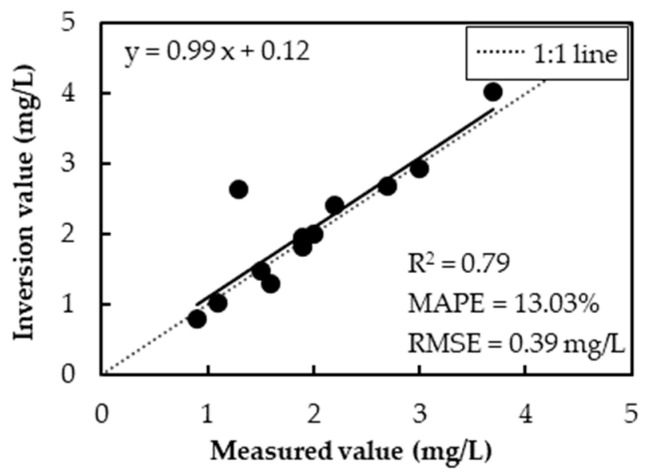
Accuracy assessment result.

The results of the model accuracy assessment are shown in Figure 5. The R^2^ of the model is 0.79, and the slope of the regression curve is 0.99, which is close to the 1:1 line, indicating a high level of fitting accuracy. Additionally, the mean absolute percentage error (MAPE) is 13.03%, and the root mean square error (RMSE) is 0.39 mg/L, suggesting that the error between the remote sensing inversion values and the measured values is relatively small. A sample with a large distance deviation is observed in Figure 5, consistent with the statistical results mentioned above, accounting for 7.69% of the total samples.

### 3.3. Temporal and Spatial Distribution Characteristics

**Figure 6:**
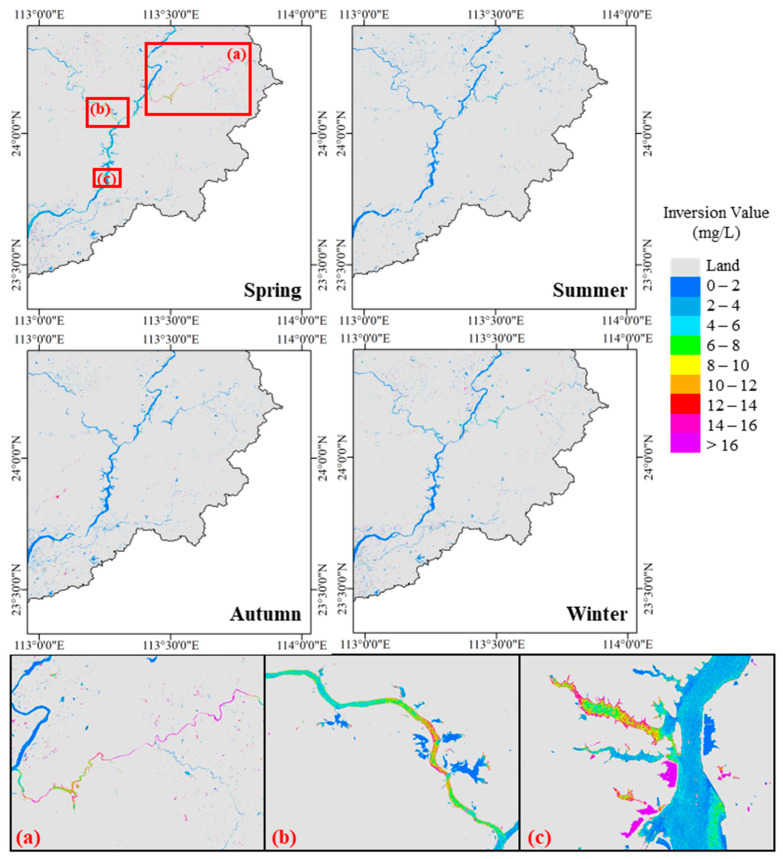
Seasonal variation of organic pollutants concentration in Feilaixia Basin during 2023: (**a**) the details of the Wengjiang River, (**b**) the details of the Lianjiang River, (**c**) the details of the Feilaixia Water Conservancy Hub.

Based on the model calculations conducted in this study, the concentrations of organic pollutants in the Feilaixia Basin in 2023 exhibited distinct seasonal variations, as shown in Figure 6. During spring (March), the concentration of organic pollutants was notably higher than in other seasons. This increase may be attributed to the combined influence of meteorological drought and intensive agricultural activities, such as spring plowing and fertilization. In contrast, summer (May) and fall (September) saw a decline in the organic pollutant concentration, indicating a partial alleviation of pollution. This reduction is likely associated with frequent heavy rainfall during these periods, which diluted pollutant concentrations and promoted water exchange. However, a slight increase in organic pollutant concentrations was observed during winter (December), potentially due to lower temperatures, reduced precipitation, and weaker hydrodynamic conditions.

Regarding spatial distribution, the main channel of the Beijiang River generally exhibited low inversion values, with organic pollutant concentrations ranging from 0 to 4 mg/L, indicating an absence of widespread organic pollution. However, higher inversion values, suggesting the presence of organic pollution, were observed in certain tributaries, such as the Wengjiang River (Figure 6a), the Lianjiang River (Figure 6b), and some reservoir bays of the Feilaixia Water Conservancy Hub (Figure 6c).

In the Wengjiang River, the remote sensing inversion values were notably high, with organic pollutant concentrations in some areas exceeding 16 mg/L. Moving from east to west along the river, the concentration gradually decreased, reaching moderate levels near the Changhu Reservoir. However, the concentration increased again until improving near the main stream. In contrast, high-value areas in the Lianjiang River were primarily concentrated downstream, with a high concentration zone in the middle section that extended toward both upstream and downstream, showing a gradual reduction in pollutant concentration. In the Feilaixia Water Conservancy Hub, elevated values were mainly found in certain reservoir bays on the west bank of the river. These highest concentrations typically occurred near the edges of the bays, with a gradual decrease toward the center. These spatial patterns are likely influenced by the distribution of pollution sources such as industrial parks, urban settlements, and aquaculture activities, which are concentrated along certain tributaries and reservoir bays.

## 4. Discussion

### 4.1. Error Analysis

According to the accuracy assessment results, a significant deviation was observed in sample NO.9. This sample is located in the middle reaches of the Longxi River, a tributary of the Beijiang River, within Shijiao Town, Fogang County, Qingyuan City. This area features a medium-sized reservoir primarily used for flood control and irrigation. According to public information from the Qingyuan Water Conservancy Bureau [57], the region experiences issues related to soil erosion. Moreover, the encroachment of vegetation and trash into the river channel negatively impacts both the natural environment and human settlements. Given these factors, it is conceivable that sample NO.9 may be impacted by environmental pollution.

This deviation can be attributed to several factors. Firstly, the measured data used in this study were obtained from automated monitoring at the monitoring station, which makes it challenging to completely eliminate the influence of factors such as instrument malfunctions and obstruction by vegetation or trash on data accuracy. During the monitoring process, instrument malfunctions may result in erroneous or missing data, while physical obstruction could disrupt the data collection, leading to unstable or inaccurate results [9,11]. Additionally, water quality changes are influenced by various factors and exhibit a certain degree of instability. Difficulties in achieving quasi-synchronization between the monitoring time and satellite transit time could also impact the comparative analysis of data [10,19]. Therefore, it is essential to carefully consider these potential impacts during data processing and result interpretation. Measures should be implemented to minimize the effects of these factors to ensure the reliability and accuracy of the study findings.

### 4.2. Influencing Factors of Spatiotemporal Distribution

According to the 2023 Qingyuan Climate Bulletin [58], the region experienced a severe meteorological drought from mid-February to mid-March, with 35 consecutive days without effective precipitation. Due to the combination of high temperatures and low rainfall, a moderate or higher meteorological drought occurred during the spring. Over the course of the year, there were ten widespread heavy rainfall events, primarily between late March and early October, with summer rainfall accounting for nearly half of the total annual precipitation. Additionally, six typhoons affected the city, predominantly between mid-July and mid-October, contributing some rainfall. Summer was characterized by hot, humid conditions and frequent heavy rainfall, while thunderstorms and typhoon-induced rains persisted into the fall. Winter saw several cold air events, with the most significant cold front occurring in late December, which was the second strongest to affect Guangdong Province since 1978.

The meteorological data indicate that climatic conditions play a significant role in the seasonal changes of organic pollutant concentrations in the Feilaixia Basin. Rainfall, in particular, is the primary factor driving these fluctuations [59,60]. It dilutes organic pollutants in the river, resulting in a seasonal pattern of higher concentrations in spring, a reduction during summer and fall, and a slight increase in winter.

Beyond seasonal factors, the spatial distribution of organic pollutants shows distinct regional trends. High concentration areas typically indicate pollution sources, and as the pollutants are transported by the river, their concentrations decrease and diffuse in a specific direction, forming a regional pattern.

Two large towns, Donghua Town and Qiaotou Town, are situated along the middle reaches of the Wengjiang River, with urban agglomerations concentrated on both sides. Additionally, a large number of industries are distributed along the middle reaches, including two large industrial parks, Dongbao Industrial Park and Yingde Overseas Chinese Industrial Park. In the downstream region of the Lianjiang River, two major towns, Shuibian Town and Lianjiangkou Town, are nearby. Similarly, near the Feilaixia Water Conservancy Hub, two large towns, Lixi Town and Feilaixia Town, are distributed, although the density of urban clusters along this section of the river is relatively low. Investigations have revealed the prevalence of small-scale farming and aquaculture in both areas, with irrigation wastewater and aquaculture waste being directly discharged into the river. Particularly in reservoir bays, where water mobility is low, organic pollutants are more likely to accumulate.

Thus, the spatial variation in organic pollutant concentrations is likely driven by human activities along the river, such as industrial and urban wastewater discharge, the use of organic pesticides, and aquaculture practices [61,62].

### 4.3. Limitations and Future Applications

Atmospheric correction is a critical step in optical satellite remote sensing for estimating water quality parameters, but it still faces significant challenges. Several simple, user-friendly integrated atmospheric correction algorithms, such as 6S (Second Simulation of Satellite Signal in the Solar Spectrum), FLASSH (Fast Line-of-Sight Atmospheric Analysis of Spectral Hypercubes), and QUAC (Quick Atmospheric Correction), have been developed [63]. While these methods are somewhat generalizable for water quality remote sensing, they still require improvement in terms of accuracy [64]. In this study, we directly extracted clear and deep water pixel data from satellite imagery as atmospheric control points to obtain the necessary parameters for atmospheric correction. The advantage of this method lies in its thorough consideration of atmospheric conditions during imaging, as well as the absorption and scattering effects of water vapor above the water surface, resulting in significant atmospheric correction for water pixel data. However, this approach does not fully account for the atmospheric components above land pixels, which leads to discrepancies in the correction of land pixels. As a result, while this method performs well in water quality remote sensing, it may introduce errors in urban remote sensing applications. In future studies, additional types of reference pixels may be introduced to improve the estimation of atmospheric optical parameters. For example, dense vegetation pixels on land could be used alongside clear deep-water pixels, enabling a more accurate atmospheric correction for both terrestrial and aquatic surfaces.

With the global advancement of inland water environmental monitoring, organic pollutant remote sensing inversion algorithms have also been evolving and improving. During the modeling process of this study, several assumptions were made to ensure the model’s effectiveness, which may introduce some degree of error to the results [65]. Future research should focus on optimizing these assumptions under various environmental conditions, thereby improving both computational efficiency and model accuracy. Additionally, this study successfully acquired the inherent optical parameters of organic pollutants and developed a model based on radiative transfer that demonstrates good regional adaptability. Future work could extend this algorithm to different inland water environments to test its generalizability and expand its application, enabling large-scale dynamic monitoring [32]. This would provide water quality managers with more accurate data for more scientific and effective decision making.

## 5. Conclusions

In this study, we present a remote sensing method for the quantitative estimation of organic pollution in inland waters. Through a combination of indoor and outdoor experiments, we obtained the key optical parameters required by the algorithm, including the absorption and scattering coefficients for organic pollutants. The algorithm was then applied to the Feilaixia Basin, and the accuracy assessment results revealed an R^2^ of 0.79, an MAPE of 13.03%, and an RMSE of 0.39 mg/L. These results indicate that the algorithm performs effectively in the quantitative estimation of the organic pollutant concentration in water bodies. Furthermore, the algorithm was used to generate a seasonal variation map of organic pollutant concentrations in the Feilaixia Basin. The findings show that the overall water quality in the basin is relatively good; certain river segments, such as the Wengjiang River and the lower reaches of the Lianjiang River, are affected by organic pollutants. Particularly in the spring, pollution levels are more severe due to the impact of precipitation.

In conclusion, this study not only advances the application of remote sensing for monitoring organic pollutants but also provides a reliable reference for water environment monitoring using remote sensing technologies. The results of this study can support environmental protection agencies and water management authorities in identifying high-risk areas, developing seasonal intervention strategies, and implementing targeted pollution control measures. The quantitative outputs and spatial distribution maps generated by the model can be directly integrated into water quality early warning systems and regulatory planning, thereby improving the scientific basis and efficiency of water resource management decisions.

## Figures and Tables

**Table 1:** The spectral information of Sentinel-2.

Band Number	Central Wavelength (nm)	Bandwidth (nm)	Spatial Resolution (m)
1	443	20	60
2	490	65	10
3	560	35	10
4	665	30	10
5	705	15	20
6	740	15	20
7	783	20	20
8	842	115	10
8b	865	20	20
9	945	20	60
10	1375	30	60
11	1610	90	20
12	2190	180	20

**Table 2:** Statistical comparison between the measured value and the remote sensing inversion value of organic pollutants.

No.	Measured Value (mg/L)	Inversion Value (mg/L)	RE
1	1.5	1.471	0.02
2	1.6	1.303	0.19
3	1.9	1.811	0.05
4	3.7	4.020	0.09
5	3.0	2.921	0.03
6	2.2	2.418	0.10
7	1.9	1.897	0.00
8	2.0	1.994	0.00
9	1.3	2.625	1.02
10	1.1	1.031	0.06
11	1.9	1.952	0.03
12	2.7	2.682	0.01
13	0.9	0.801	0.11
**Min**	0.9	0.801	0.00
**Max**	3.7	4.020	1.02
**Average**	2.0	2.071	0.13

## Data Availability

The original contributions presented in this study are included in the article. Further inquiries can be directed to the corresponding author.
